# Vitrification of human ovarian tissue: a practical and relevant alternative to slow freezing

**DOI:** 10.1186/s12958-015-0065-5

**Published:** 2015-06-25

**Authors:** Sandra Sanfilippo, Michel Canis, Johan Smitz, Benoît Sion, Claude Darcha, Laurent Janny, Florence Brugnon

**Affiliations:** Centre international de chirurgie endoscopique, Clermont-Ferrand, France; CHU Clermont-Ferrand, CHU Estaing, Pôle gynécologie obstétrique et reproduction humaine - Assistance médicale à la procréation - CECOS, Clermont-Ferrand, France; Follicle biology laboratory, Vrije Universiteit Brussel, B - 1090 Jette, Belgium; Laboratoire de physiologie, Université Clermont 1, UFR Pharmacie, Inserm U1107 NEURO-DOL, Clermont-Ferrand, France; CHU Clermont-Ferrand, CHU Gabriel Montpied, Anatomie et cytologie pathologiques, Centre de biologie, Clermont-Ferrand, France; Faculté de médecine, CNRS-UMR 6293/INSERM U1103, Laboratoire de génétique, reproduction et développement, Université d’Auvergne, Clermont-Ferrand, France

**Keywords:** Cryopreservation, Human ovarian tissue, Vitrification, Conventional slow-freezing, Follicle morphology, DNA fragmentation

## Abstract

**Background:**

Cryopreservation of ovarian tissue can be used to preserve the fertility of patients who are about to receive treatment(s) that could compromise their future ovarian function. Here we evaluate the effectiveness of a vitrification protocol by carrying out a systematic comparison with a conventional slow-freezing method on human ovarian tissue.

**Methods:**

Human ovarian samples (mean age 28.0 ± 1.1 years) were processed in parallel for each cryopreservation procedure: vitrification and slow-freezing. Following warming/thawing, histological observations and a TUNEL assay in ovarian follicles were performed and compared to unfrozen control.

**Results:**

Both cryopreservation protocols gave comparable histological outcomes. Percentage of intact follicles was 83.6 % following vitrification in a 1.5 M 1,2-propanediol (PrOH), 1.5 M ethylene glycol (EG) and 0.5 M raffinose solution, 80.7 % after slow-freezing in 1.5 M PrOH and 0.025 M raffinose, and 99.6 % in fresh tissue. Follicle density was unchanged by vitrification (0.6 follicles/mm2) or slow-freezing (0.5 follicles/mm2) compared to fresh tissue (0.7 follicles/mm2). Percentage of follicles with DNA fragmentation was not statistically different in vitrified (20.8 %) or slow-frozen (31.3 %) tissues compared to the unfrozen control (35.0 %). There was no difference in proportion of stroma cells with DNA fragmentation in vitrified (6.4 %) and slow-frozen (3.7 %) tissues compared to unfrozen tissue (4.2 %).

**Conclusions:**

This vitrification protocol enables good preservation of ovarian quality post-warming. The evaluation of endocrine function after vitrification need to be perform in a higher cohort to evaluate if this protocol may offer a relevant alternative to conventional slow-freezing for the cryopreservation of human ovarian tissue.

## Background

Cryopreservation of ovarian tissue followed by auto-transplantation is a promising method for fertility preservation in girls and young women at risk of premature ovarian insufficency as a result of anti-cancer treatment when ovarian stimulation is not possible [1, 2]. Cryopreservation of ovarian tissue can be performed by slow-freezing or vitrification. Slow-freezing has resulted in 37 live births worldwide after orthotopic transplantation [3, 4]. However, two major issues with slow-cooling protocols are that they are time-consuming and often require costly equipment. We recently addressed the first issue by developing a serum-free 1,2-propanediol (PrOH), raffinose-based solution supplemented with antioxidants that gave promising results in terms ovarian integrity and functionality, even when used in combination with a faster cooling program than the usual one [5, 6]. Over the last decade, numerous studies have investigated vitrification as an alternative to conventional slow-freezing for ovarian tissue [7], and different vitrification solutions and methods, mostly adapted from blastocyst and oocyte vitrification, have been applied. Live offspring have been born from vitrified mouse ovarian preantral follicles matured *in vitro*, and orthotopic autografting of vitrified/warmed sheep hemi-ovaries has led to the birth of four lambs [8–10]. In humans, two live births were recently reported after ovarian tissue vitrification followed by *in vitro* activation of dormant follicles in patients with primary ovarian insufficiency [11]. The issue of whether vitrification is superior to slow-freezing for cryopreserving human ovarian tissue remains unresolved. Rahimi *et al*. observed a higher percentage of apoptotic cells in vitrified human ovarian tissues after grafting compared to slow-frozen tissues [12], and Oktem *et al*. showed higher primordial follicle density and viability after slow-freezing compared to vitrification [13]. However, other studies have failed to find any difference between these two cryopreservation procedures in terms of the proportion of morphologically intact follicles and proportion of apoptotic cells [14, 15]. These discrepant conclusions likely reflect either heterogeneity in the cryopreservation protocols applied, which ovarian components may be particularly sensitive to, and/or disparities in the methods employed to evaluate ovarian tissue quality. Limited access to donated ovarian tissue places limits to comparative studies between vitrification and slow-freezing methods, and few studies have compared the effects of these two methods on ovarian tissue from the same patient. Here we investigated follicle integrity following vitrification with a novel PrOH, ethylene glycol (EG) and raffinose-based procedure. The efficiency of this vitrification procedure was evaluated by carrying out a systematic comparison with our earlier PrOH and raffinose-based slow-freezing protocol.

## Methods

Unless otherwise indicated, all products were purchased from Sigma-Aldrich (France).

### Ovarian tissue collection

**Fig. 1:**
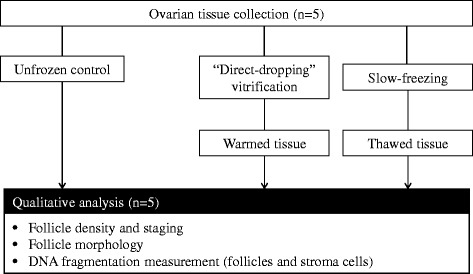
Experimental design. Follicle morphology and staging as well as DNA fragmentation measurements from warmed and thawed ovarian tissue were assessed in parallel and compared against unfrozen controls (*n* = 5)

This study was approved by the regional research ethics committee (Comité Consultatif des Personnes se Prêtant à la Recherche Biomédicale d’Auvergne, Project AU 419, 07/03/2001). From 1 October 1 2013 to 1 May 2014, ovarian cortical samples from 5 patients were collected during endoscopic surgery for benign cysts, after signed informed consent. Mean age of the women was 28.0 **±** 1.1 (SD) years. For each patient, a piece of ovarian cortex overlying the cyst was excised with scissors and without electrocoagulation. The specimens were immediately immersed in the holding “medium A” at 4 °C and transported to the laboratory on ice, as previously described [16]. “Medium A” was composed of: NaCl (94.7 mM), KCl (4.8 mM), MgSO_4_ (0.8 mM), NaH_2_PO_4_ (1.0 mM), NaHCO_3_ (25.0 mM), CaCl_2_ (1.8 mM), sodium lactate (21.3 mM), sodium pyruvate (0.3 mM), D-glucose (5.5 mM), L-glutamine (25.0 mM), taurine (0.5 mM), and 0.5 % of human serum albumin (Vitrolife Sweden AB, Sweden). The cortex was cut into fifteen to twenty 1 × 1 × 5-mm slices. For each patient, 5 tissue pieces were processed for light microscopy and TUNEL assay (unfrozen control), and the remaining specimens were randomly divided into vitrification and slow-freezing groups. Samples from any one patient were processed for each cryopreservation procedure at the same time, warmed/thawed and analyzed in parallel (Fig. 1).

### Vitrification/warming procedure

Our vitrification procedure consisted of three incubation steps in solutions with increasing concentrations of permeable cryoprotectants (CPAs) dissolved in “Medium A” supplemented with 0.5 % HSA, HEPES (21.8 mM) and glycine (50.0 mM). Ovarian slices were sequentially immersed for 5 min in the solution at 25 % CPAs [0.37 M PrOH, 0.37 M EG], 5 min at 50 % CPAs [0.75 M PrOH, 0.75 M EG] and 10 min at 100 % CPAs [1.5 M PrOH, 1.5 M EG]. At the third step, the vitrification solution was supplemented with 0.5 M raffinose. The first and second incubation steps were performed at room temperature, while at the higher CPA concentrations, the samples were incubated at +4 °C. The slices were then dropped directly into liquid nitrogen, together with 20 μL of 100 % vitrification solution as previously described [17]. Afterwards, the vitrified tissue was transferred to a pre-cooled 1.8 mL sterile cryovial (Nunc, Fisher Bioblock Scientific, France) and stored in liquid nitrogen.

For the warming procedure, cryovials were immersed in a 37 °C water bath for 2 min and the CPAs were diluted by transferring the ovarian pieces through decreasing concentrations of vitrification solution (50 % and 25 %) at 37 °C. Each step of dilution protocol lasted 5 min. Each slice was then washed twice in “Medium A” + 0.5 % HSA for 5 min at 37 °C.

### Slow-freezing procedure

The cryopreservation medium B consisted of “Medium A” supplemented with 0.5 % HSA, HEPES (21.8 mM), glycine (50.0 mM), PrOH (3.0 M) and raffinose (0.05 M). “Medium B” was added in three steps to “medium A” containing the ovarian slices, to a final concentration of 1:1 (v/v) under gentle agitation [1.5 M PrOH and 0.025 M raffinose]. After 15 min of equilibration at 4 °C, each slice was transferred to a sterile cryovial containing 1.5 mL of cryoprotective solution and loaded into a programmable freezer (Minicool 40 PC, Air Liquide, France) held at 4 °C. The cooling rate was −2 °C/min from 4 °C to −11 °C, at which point temperature nucleation was induced by semi-automatic seeding. The temperature was then lowered to −40 °C at −2 °C/min and from −40 °C to −150 °C at −10 °C/min. Finally, the cryovials were plunged into liquid nitrogen for storage.

For the thawing procedure, cryovials were immersed in a 37 °C water bath for 2 min and the cryoprotective solution containing the ovarian slices was diluted in two 5-min steps with the basal “medium A” + 0.5 % HSA, at 37 °C. Each slice was then washed twice in “medium A” + 0.5 % HSA for 5 min at 37 °C.

### Histological examination

Fresh as well as warmed/thawed ovarian fragments were fixed overnight in alcohol/formalin/acetic acid at 4 °C, then paraffin-embedded and cut into 4 μm serial sections. Eight consecutive sections were mounted *per* slide and every second slide was deparaffinized, hydrated and stained with hematoxylin and eosin. The sections were observed by light microscopy at ×400 magnification to establish the development stage of the follicles according to Gougeon’s criteria: primordial (flattened granulosa cells (GCs)), intermediary (mixture of flattened and cuboidal GCs), primary (single layer of cuboidal GCs) and secondary (two or more layers of GCs) [18]. In the result section, primordial and intermediary follicles have been pooled into one group and termed as “resting follicles”. Follicle morphology was evaluated on the basis of previously-described parameters [19]. Follicles were classified as intact if there were no overt signs of oocyte and GC degeneration. The basement membrane of the follicle had to be intact and attached to the GC layer and the oocyte had to be in contact with its surrounding GCs. The follicles were regarded as degenerated if they contained an intact oocyte but showed more than 50 % of the following signs: detachment of the oocyte from surrounding GCs and/or vacuolization of ooplasm and/or partially-degenerated GCs and/or detachment of the basal membrane.

To estimate follicular density, the histological sections were digitized *via* a Matrox Meteor MC/4 card (Samba technologies, France) coupled to a Sony 3CCD DXC 950P color camera (Sony Corp., Japan). The area of the sections was determined by delineation of the tissue boundary using IPS 32 version 4.27 software (Samba Technologies). Follicle density in the ovarian cortex was calculated as total number of follicles divided by area of the cortex analyzed (mm2).

### Measurement of DNA fragmentation

DNA fragmentation in the follicles was detected using the *In situ* Cell Death Detection Kit (Roche, France) according to the manufacturer’s protocol. After rehydration and permeabilization, the sections were incubated with a labeling solution containing dUTP and enzyme solution (Terminal deoxynucleotidyltransferase, Tdt) for 1 h at 37 °C. After counterstaining with Hoechst 33258 (Invitrogen, France), the tissue sections were observed by fluorescence microscopy. A negative control was carried out by omitting Tdt from the reaction mixture. A positive control was performed by applying DNAse treatment. Follicles with positive TUNEL staining of the oocyte and/or ≥ 50 % of the GCs were considered as positive. The proportion of TUNEL-positive stroma cells was evaluated on three fields at ×400 magnification *per* section. Images were captured using a Nikon DSFI-1 digital camera (Nikon, Japan).

### Statistical analysis

All data were summarized using frequency counts and percentages. For the statistical analysis, a generalized linear mixed model was built with patient as random factor. Comparisons between groups (unfrozen, vitrification and slow-freezing) were corrected for simultaneous hypothesis testing according to Tukey-Kramer. A *p*-value < 0.05 was considered statistically significant.

## Results

### Follicle distribution and morphology

**Fig. 2:**
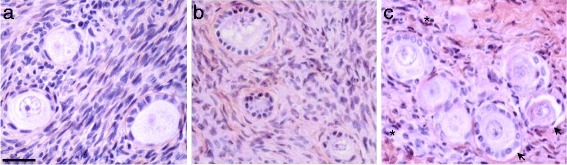
Histologic assessment of unfrozen, vitrified and slowly frozen human ovarian follicles. Sequentially hematoxylin and eosin stained human ovarian tissues from the unfrozen control (**a**), vitrification (**b**) and slow-freezing (**c**) groups. Tissues from the three groups are mainly composed of follicles at resting and primary stages. Well-preserved follicles exhibited intact nuclear and cellular membranes, uniform ooplasm and a prominent nucleus of the oocyte. Note two degraded follicles (black arrows) in the tissue cryopreserved according to the slow-freezing method (**c**), showing over 50 % oocyte detachment from surrounding GCs. Surrounding stroma in unfrozen tissue (**a**) and vitrified (**b**) tissues was compact, and stromal cells had spindle-shaped nuclei. Note increased numbers of pycnotic cells (black stars) and empty areas in the stromal tissue after slow-freezing (**c**). Scale bar = 20 μm

In total, 249 follicles were analyzed in fresh tissue processed before cryopreservation, 482 after vitrification and 374 after slow-freezing (*n* = 5 patients). After vitrification, follicle density (0.6 follicles/mm2) was well maintained and not statistically different to fresh (0.7 follicles/mm2) or slow-cooled (0.5 follicles/mm2) tissues (*p* > 0.05). The results are shown in Table 1. In the fresh group, 81.5 % of these follicles were classified as resting, 15.7 % as primary and 2.8 % as secondary. After vitrification, 96.9 % of follicles were classified as resting, 2.3 % as primary and 0.8 % as secondary. In the slow-freezing group, 97.6 % of follicles were classified as resting and 2.3 % as primary after thawing. Comparative analysis showed that the unfrozen group had a statistically higher number of follicles in progressed stages (primary and secondary) compared to the vitrification and slow-freezing groups. However, there was no significant difference in distribution of follicles of different developmental stages between the two cryopreservation groups (*p* > 0.05) (Table 1). In unfrozen tissue (Fig. 2a), follicles displayed close adherence between oocyte and GCs. The oocytes had a homogenous cytoplasm, and no vacuolization was observed. Stromal cells had spindle-shaped nuclei and no interstitial edema was present. In the vitrified follicles (Fig. 2b), the close contact between oocyte and GCs was well maintained. The vitrified follicles showed intact nuclear and cellular membranes and a uniform oocyte cytoplasm. Quality of the stroma was compact, morphologically normal, and comparable to that in unfrozen control. In the slow-freezing group, partial or total detachment of the basal membrane was the main morphological alteration observed in follicles (Fig. 2c, black arrows). The surrounding stroma showed empty spaces and disorganized architecture (Fig. 2c, black stars). Overall analysis of follicle morphology showed that 83.6 % and 80.7 % of follicles remained morphologically intact in the vitrification and slow-freezing groups, respectively, and there were no significant differences between these groups (Fig. 2). However, the proportions of morphologically intact resting follicles were significantly reduced both after vitrification (84 %) and slow-freezing (80.3 %) compared to fresh tissue (100 %) (both *p* < 0.01).

**Table 1:** - Impact of cryopreservation protocol (vitrification versus slow-freezing) on morphology of ovarian follicles according to developmental stage

		Number of resting follicles (%)		Number of primary follicles (%)		Number of secondary follicles (%)	
Group	Total number of follicles	Total	Intact	Total	Intact	Total	Intact
Unfrozen	249	203 (81.5 %)	203 (100 %)	39 (15.7 %)	38 (97.4 %)	7 (2.8 %)	7 (100 %)
Vitrification	482	467*** (96.9 %)	392*** (84 %)	11** (2.3 %)	9 (81.8 %)	4* (0.8 %)	2 (50 %)
Slow-freezing	374	365*** (97.6 %)	293*** (80.3 %)	9** (2.4 %)	9 (100 %)	0* (0 %)	_

Data are expressed as numbers and proportions [n (%)]. *0.01 < *p* < 0.05, **0.001 < *p* < 0.01, ***p < 0.001 compared to the unfrozen control

### Assessment of DNA fragmentation

**Fig. 3:**
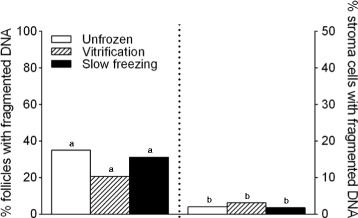
DNA fragmentation in follicles. Histograms presenting proportion of TUNEL-positive follicles (left panel) and stroma cells *per* high power field before (white plots) and after cryopreservation using vitrification (black hatched plots) *versus* slow freezing methods (black plots) (*n* = 5 patients). Pairwise comparisons between unfrozen, vitrification and slow-freezing groups were performed using a Tukey-Kramer test. ^a,b^ No difference among the three groups (^a^ *p* > 0.05, ^b^ *p* > 0.05)

*In situ* DNA fragmentation was assessed in the follicles using the TUNEL assay. For this measurement, a total of 155 follicles were analyzed: 62 before cryopreservation, 64 after vitrification, and 29 after slow-freezing. Pairwise comparisons showed no significant difference in proportion of follicles with DNA fragmentation in cryopreserved (vitrification: 20.8 %; slow-freezing: 31.3 %) *versus* fresh tissues (35 %) (*p* > 0.05 respectively). Moreover, no statistical difference was found between the two cryopreservation groups (*p* > 0.05) (Fig. 3 left panel). DNA fragmentation was assessed in 1536 fresh, 1032 warmed and 1076 thawed stroma cells. We found no significant increase in percentage of stroma cells with DNA fragmentation both after vitrification (6.4 %) and slow-freezing (3.7 %) compated to unfrozen control (4.2 %) (*p* > 0.05 respectively). Although stoma cells in warmed tissue tended to show increased DNA fragmentation, the vitrification and slow-freezing groups were not statistically significantly different (*p* > 0.05) (Fig. 3 right panel).

## Discussion

Vitrification is a rapid and simple technique which has recently shown real prospects for the cryopreservation of heterogeneous biologic tissues such as ovarian cortex [20]. In order to minimize cryoprotectant toxicity without affecting vitrification properties, relatively low concentrations of different cryoprotectants can be combined [21]. Here we chose a combination of PrOH and EG as CPAs based on successful results of oocyte and embryo vitrification [22]. Moreover, a recent study reported better results on mouse ovarian tissue with the combination [20 % EG (v/v) + 20 % PrOH (v/v)] than with combinations [20 % EG (v/v) + 20 % DMSO (v/v)] or [20 % PrOH (v/v) + 20 % DMSO (v/v)] in terms of preserving follicle integrity and functionality [23]. Raffinose was used as non-penetrating cryoprotectant in order to increase water withdrawal from cells and decrease cryoprotectant exposure time [24]. de la Peña *et al*. reported successful vitrification of preantral follicles from mice using a cryoprotective solution supplemented with 6 M EG and 0.3 M raffinose [8]. There is currently still no commercialized carrier system suitable for vitrification of human ovarian tissue in medical practice. To achieve ultra-rapid cooling, we used the “direct dropping” technique [17]. The minimal volume of vitrification solution surrounding the samples could maximize the cooling rate and reduce the toxicity of the vitrification solution with less-concentrated cryoprotectants. After vitrification of human ovarian cortex using this carrier-less system, Amorim *et al*. reported a significantly higher proportion of morphologically intact follicles compared to solid surface vitrification (SSV) and plastic straw methods [25].

The results of our study show that vitrification preserves follicle and stroma morphology as well as the slow-freezing method. Our data are in agreement with previous reports where systematic comparisons between the two cryopreservation procedures were carried out on human ovarian tissue [19, 15]. Histomorphometric analysis showed that follicular densities were comparable between warmed and thawed tissues. Our vitrification protocol did not increase the percentages of follicles and stroma cells with DNA fragmentation after warming. Ovarian cortex was thus vitrified without the subsequent irreversible DNA damage potentially arising from apoptosis and/or oxidative stress activation [12, 26]. In a previous study, Xiao *et al*. reported a significant increase in TUNEL-assessed DNA fragmentation in human follicles and stroma cells after vitrification by direct contact of ovarian cortex with liquid nitrogen [27]. Supplementation of the collection and vitrification medium with taurine and L-glutamine, which have been found to play an antioxidant role by reducing cryopreservation-induced oxidative stress, might explain the superiority of our vitrification procedure over the above-mentioned report [28, 29, 6]. Taken together, our findings indicate that human ovarian tissue maintains similar quality after vitrification or slow-freezing.Due to limited access to samples of human ovarian tissue, only 5 patients were enrolled in the present study. However, the originality of our study lies in the fact that the vitrification and slow-freezing protocols were systematically compared by analyzing ovarian tissue obtained from the same patient. Although the total number of follicles recovered from these samples was significantly higher than in other studies that have included 10 to 15 patients more, it would be prudent to complete our study with a higher number of patients [19, 14]. We recently developed a low-attachment 3D culture system that has yielded encouraging results in terms of developmental capacity of cryopreserved human follicles [6]. It would therefore be of great interest to use this new *in vitro* model to gain deeper insights into the impact of the vitrification procedure on ovarian function. From this standpoint, an evaluation of 17β-oestradiol and progesterone production in culture would be required as an indicator of follicular growth and secondary follicle viability. Although reported as the most successful vitrification method, the “direct dropping” technique used in this study presupposes direct contact with liquid nitrogen, which is a potential source of microbial contamination [30]. Since the manufacturers have not yet come up with a closed system suitable for ovarian tissue, application of our vitrification procedure in clinical practice requires the use of sterilized liquid nitrogen. Despite its technical constraints (costly, time-intensive…), UV-sterilization of liquid nitrogen remains the main way to guarantee “safe vitrification” in medical practice.

## Conclusions

This study shows that human ovarian tissue retains a comparable morphological appearance whether after vitrification or slow-freezing. These promising results raise prospects for using this vitrification protocol in routine practice as a relevant alternative to slow-freezing for cryopreserving human ovarian tissue.

## Abbreviations

CPA: Cryoprotective agent
EG: Ethylene glycol
GC: Granulosa cell
HSA: Human serum albumin
PrOH: 1,2-Propanediol
